# Centenarian longevity had inverse relationships with nutritional status and abdominal obesity and positive relationships with sex hormones and bone turnover in the oldest females

**DOI:** 10.1186/s12967-021-03115-7

**Published:** 2021-10-18

**Authors:** Shihui Fu, Ping Ping, Yulong Li, Bo Li, Yali Zhao, Yao Yao, Pei Zhang

**Affiliations:** 1 Cardiology Department, Hainan Hospital of Chinese People’s Liberation Army General Hospital, Sanya, China; 2 Department of Geriatric Cardiology, Chinese People’s Liberation Army General Hospital, Beijing, China; 3 Pharmacy Department, Chinese People’s Liberation Army General Hospital, Beijing, China; 4 Central Laboratory, Hainan Hospital of Chinese People’s Liberation Army General Hospital, Sanya, China; 5 Center for Healthy Aging and Development Studies, National School of Development, Peking University, Beijing, China; 6 School of Life Science, Beijing Institute of Technology, Beijing, China

**Keywords:** Abdominal obesity, Bone turnover, Centenarian longevity, Nutritional status, Sex hormones

## Abstract

**Objective:**

The number of older people is estimated to increase from 524 million in 2010 to 1.5 billion in 2050. The factors and models of human longevity and successful aging are questions that have intrigued individuals for thousands of years. For the first time, the current study was designed to investigate the relationships between sex hormones, bone turnover, abdominal obesity, nutritional status and centenarian longevity in the oldest females.

**Methods:**

The China Hainan Centenarian Cohort Study was performed in 18 cities and counties of Hainan Province using standard methodology in 500 centenarian females and 237 oldest females aged between 80 and 99 years.

**Results:**

Centenarians were inversely associated with the geriatric nutritional risk index [Exp(B) (95% CI): 0.901 (0.883–0.919)] and abdominal obesity [Exp(B) (95% CI): 0.719 (0.520–0.996)] and positively associated with prolactin [Exp(B) (95% CI): 1.073 (1.044–1.103)], progesterone [Exp(B) (95% CI): 44.182 (22.036–88.584)], estradiol [Exp(B) (95% CI): 1.094 (1.071–1.119)], osteocalcin [Exp(B) (95% CI): 1.041 (1.028–1.054)], β-crossLaps [Exp(B) (95% CI): 63.141 (24.482–162.848)] and parathyroid [Exp(B) (95% CI): 1.022 (1.013–1.031)] hormone levels (P < 0.05 for all). The geriatric nutritional risk index and abdominal obesity were inversely associated with luteinizing hormone [β coefficient (95% CI): − 0.001 (− 0.002 to 0.001)]; Exp(B) (95% CI): 0.985 (0.974–0.996)], follicle-stimulating hormone [β coefficient (95% CI): 0.000 (− 0.001 to 0.000)]; Exp(B) (95% CI): 0.990 (0.985–0.996)], osteocalcin [β coefficient (95% CI): − 0.001 (− 0.001 to 0.000)]; Exp(B) (95% CI): 0.987(0.977–0.997)] and β-crossLaps [β coefficient (95% CI): − 0.100 (− 0.130 to 0.071)]; Exp(B) (95% CI): 0.338 (0.166–0.689)] levels (P < 0.05 for all).

**Conclusions:**

Centenarian longevity had inverse relationships with nutritional status and abdominal obesity and positive relationships with sex hormones and bone turnover. Nutritional status and abdominal obesity had inverse relationships with sex hormones and bone turnover. Increased sex hormones and bone turnover may be representative of centenarian longevity. Optimizing nutritional status and avoiding abdominal obesity may increase sex hormones and bone turnover and promote centenarian longevity and successful aging.

## Introduction

The number of older people is estimated to increase from 524 million in 2010 to 1.5 billion in 2050 [1]. Among them, centenarians succeed in living beyond the typical life expectancy and are considered to experience successful aging and longevity [2]. The factors and models of human longevity and successful aging are questions that have intrigued individuals for thousands of years [3]. Centenarians may represent a prototype of human longevity and successful aging, and the oldest population, including centenarians, should be considered the best population to help address these questions [4]. Centenarians may have enhanced beneficial factors and/or weakened detrimental factors based on certain models [5]. Studies comparing centenarians and other oldest individuals could identify the factors and relationships related to centenarian longevity and provide valuable information for understanding centenarian longevity and achieving successful aging [6].

Excessive nutritional intake leads to abdominal obesity, and abnormal bone turnover can result in disabilities, falls and fractures. The risk of these conditions increases with age and particularly affects the oldest members of the population [7, 8]. Older females are prone to having less nutritional intake and longer life expectancy than are older males [9]. Therefore, sex hormones may be related to bone turnover, nutritional status, and abdominal obesity in the oldest population. Previous studies on these relationships have been performed using average adults or older people in Western countries [10]. However, ethnicity has significant effects on these relationships, and virtually no reports have been published regarding the oldest population [11]. Sex hormones, bone turnover, nutritional status, and abdominal obesity may be factors related to centenarian longevity, and centenarian longevity may be based on relationships between sex hormones, bone turnover, nutritional status, and abdominal obesity [12]. However, the evidence base for the relationships of these variables with centenarian longevity and successful aging is very limited and less convincing, requiring additional studies from different parts of the world.

**Fig. 1:**
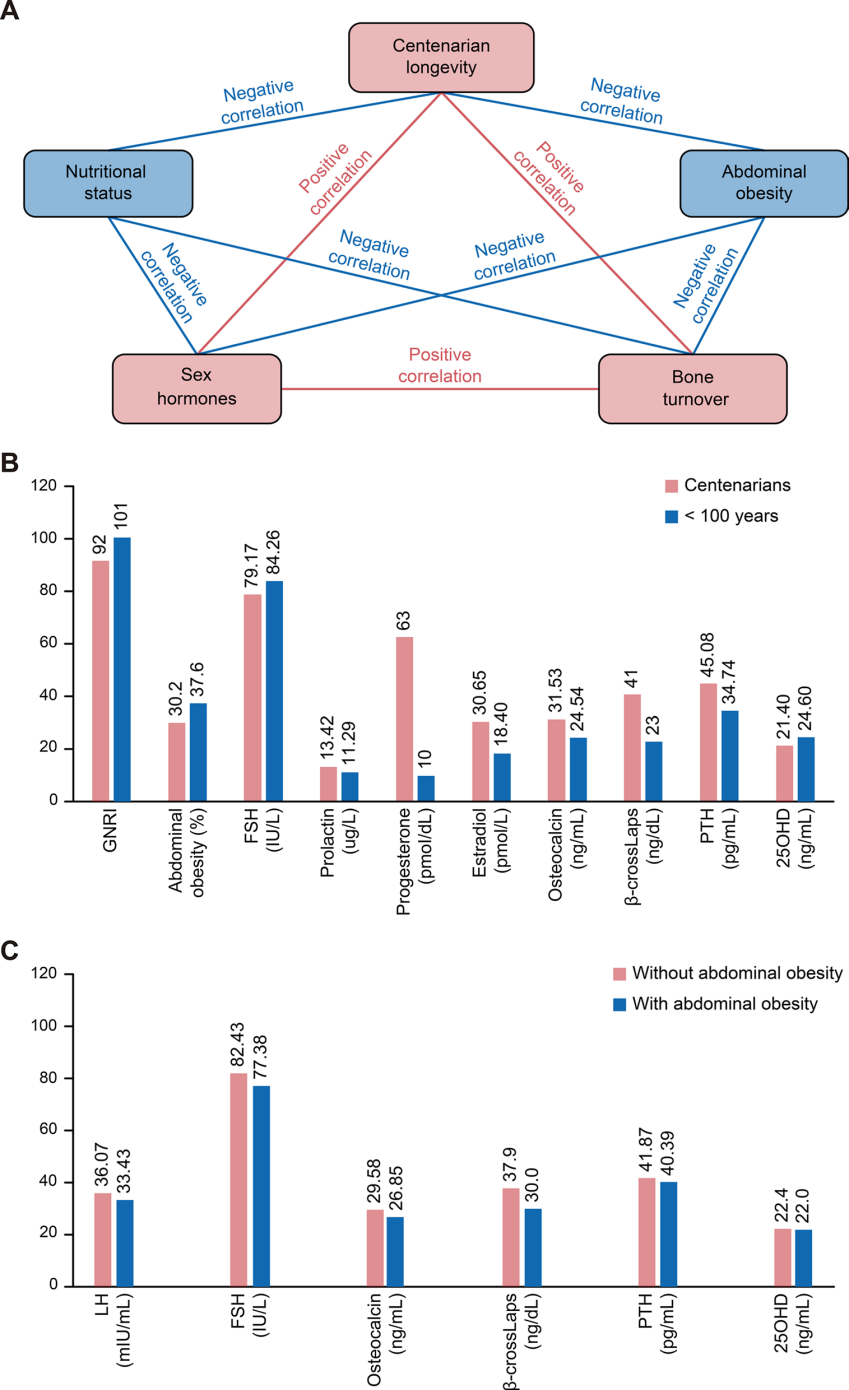
Relationships between sex hormones, bone turnover, abdominal obesity, nutritional status, and centenarian longevity in the oldest females. **A** Relationship network between sex hormones, bone turnover, abdominal obesity, nutritional status, and centenarian longevity in the Chinese oldest females; **B** different characteristics between centenarian females and other oldest females aged 80–99 years; **C** different characteristics between oldest females with and without abdominal obesity

Hainan Province is an area with high levels of longevity; in fact, it has the highest population density of centenarians in China. Therefore, the China Hainan Centenarian Cohort Study (CHCCS) provides a considerable population-based sample of centenarians [13]. For the first time, the current study using data from the CHCCS was designed to investigate the relationships between sex hormones, bone turnover, abdominal obesity, nutritional status and centenarian longevity in the oldest females (Fig. 1A).

## Materials and methods

### Study population

As a population-based study, the CHCCS was performed in 18 cities and counties of Hainan Province, China. Its profile has been described previously [13]. All participants were identified by the National Civil Registry provided by the Hainan Civil Affairs Bureau. From July 2014 to October 2017, a total of 500 centenarian females and 237 females aged 80–99 years participated in the current study and completed full and essential analyses. Considering the focus on centenarian longevity and sex hormones, the current study specifically enrolled the oldest females, including centenarians. Age was ascertained from national identification cards. This study incorporated the following inclusion criteria: (1) participants were at least 80 years old; (2) participants volunteered to join the study and provided written informed consent; and (3) participants were conscious and were able to complete home interviews, physical examinations, and blood analyses. Participants were excluded if personal identity information was not complete, identification cards showed an age < 80 years, or participants refused to comply with study requirements, including the collection of blood samples. No participants received vitamin D, exogenous steroids, or other treatments that could affect their sex hormones and bone turnover. No centenarians with oophorectomies participated in the current study. The current study was carried out based on the approval of the Ethics Committee of the Hainan Hospital of the Chinese People’s Liberation Army General Hospital (Sanya, Hainan; Number: 301hn11201601). All females provided written informed consent prior to the start of this study.

### Standard procedures

Home interviews, physical examinations, and blood analyses were carried out following standard procedures by a research team from the Hainan Hospital of the Chinese People’s Liberation Army General Hospital. This interdisciplinary research team included internists, geriatricians, cardiologists, endocrinologists, nephrologists, and nurses. All research team members were well trained according to a unified standard. Based on recommendations from the World Health Organization, weight was measured twice on a digital scale while participants wore light clothing without shoes, height was measured twice using a wall-mounted tape with participants standing without shoes, and waist circumference (WC) was measured twice using a soft tape at the midpoint between the last rib and the iliac crest [14]. Based on Chinese guidelines on the prevention and control of obesity, the presence of abdominal obesity was established when WCs were ≥ 85 cm for males and ≥ 80 cm for females [15]. Blood samples were collected and transported in chilled biotransport containers (4 °C) to the central laboratory within 4 h. Serum levels of sex hormones and bone turnover were analyzed with enzymatic analyses (Roche Products Ltd., Basel, Switzerland) on a fully automatic biochemical autoanalyzer (Cobas c702; Roche Products Ltd., Basel, Switzerland). All analyses were performed by qualified technicians without knowledge of any clinical data. Ideal female weight was calculated using the following formula [16]:

$$ {\text{height}}\;{\text{in}}\;{\text{centimeters}}\;\left( {\text{H}} \right) - 100 - \left[ {\left( {{\text{H}} - 150} \right)/2.5} \right]. $$

The geriatric nutritional risk index (GNRI) was calculated to evaluate nutritional status using the following formula [16]:

$$ \left[ {1.489 \times {\text{albumin}}\left( {{\text{g}}/{\text{L}}} \right)} \right] \, + \, \left[ {41.7 \times {\text{weight}}/{\text{ideal}}\;{\text{weight}}} \right]. $$

### Statistical analyses

Data were analyzed using the Statistical Package for Social Sciences, Ver. 17 (Chicago, IL, USA). Data were described using means and standard deviations (continuous variables with normal distributions), medians and interquartile ranges (continuous variables with skewed distributions), and numbers and percentages (categorical variables). Characteristic comparison was performed between females aged between 80–99 years and ≥ 100 years using Student’s t-tests for continuous variables with normal distributions, Mann–Whitney U tests for continuous variables with skewed distributions, and chi-square tests for categorical variables. Logistic regression was applied to analyze the relationships of centenarians with GNRI, abdominal obesity, sex hormones and bone turnover, and logistic regression was applied to analyze the relationships of abdominal obesity with sex hormones and bone turnover. Linear regression was applied to analyze the relationships of GNRI with sex hormones and bone turnover and the relationships of bone turnover with sex hormones. A two-tailed P < 0.05 was regarded as statistically significant.

## Results

### Characteristic comparison between centenarian females and females aged 80–99 years

Characteristic comparison between centenarian females and other oldest females aged 80–99 years is shown in Table 1. Centenarian females had significantly lower levels of height, weight, WC, serum albumin, and GNRI than did females aged 80–99 years (P < 0.05 for all). The proportion of abdominal obesity was significantly lower in centenarian females than in females aged between 80 and 99 years (P < 0.05). Centenarian females had significantly lower levels of follicle-stimulating hormone (FSH) and significantly higher levels of prolactin, progesterone, and estradiol than did females aged 80–99 years (P < 0.05 for all). Centenarian females had significantly higher levels of osteocalcin, β-crossLaps, and parathyroid hormone (PTH) and significantly lower levels of 25-hydroxyvitamin D3 (25(OH)D) than did females aged 80–99 years (P < 0.05 for all). Different characteristics between centenarian females and other oldest females aged 80–99 years are shown in Fig. 1B.

**Table 1:** Characteristics of all females aged 80–99 and ≥ 100 years

Characteristics	80–99 years (n = 237)	Centenarians (n = 500)	P-value
Age(year)^a^	86 ± 4.9	103 ± 2.9	< 0.001
Nutritional status			
Height (cm)^a^	145 ± 7.2	143 ± 7.7	< 0.001
Weight (kg)^a^	42.6 ± 9.1	36.4 ± 8.0	< 0.001
Serum albumin (g/dL)^a^	4.23 ± 0.31	3.90 ± 0.38	< 0.001
GNRI^a^	101 ± 9.31	91 ± 9.60	< 0.001
Waist circumference (cm)^a^	78 ± 10.1	75 ± 9.6	0.003
Abdominal obesity (%)	89 (37.6)	151 (30.2)	0.047
Sex hormones			
LH (mIU/mL)^a^	36.71 ± 12.98	37.93 ± 15.40	0.263
FSH (IU/L)^a^	87.44 ± 29.62	81.65 ± 30.17	0.015
Testosterone (nmol/L)^a^	0.50 ± 0.97	0.50 ± 0.53	0.993
Prolactin (μg/L)^a^	12.44 ± 6.52	17.18 ± 17.10	< 0.001
Progesterone (nmol/L)^a^	0.26 ± 0.27	0.75 ± 0.62	< 0.001
Estradiol (pmol/L)^a^	20.51 ± 14.78	41.54 ± 29.53	< 0.001
Bone turnover			
Osteocalcin (ng/mL)^a^	26.84 ± 12.81	35.65 ± 19.21	< 0.001
β-crossLaps (ng/mL)^a^	0.28 ± 0.18	0.46 ± 0.26	< 0.001
PTH (pg/mL)^a^	39.61 ± 21.14	49.46 ± 25.95	< 0.001
25OHD (ng/mL)^a^	24.20 ± 7.23	22.22 ± 8.14	0.001

^a^Median (interquartile range), GNRI: geriatric nutritional risk index; LH: luteinizing hormone; FSH: follicle-stimulating hormone; PTH: parathyroid hormone; 25(OH)D: 25-hydroxyvitamin-D3

### Relationships of nutritional status, sex hormones, and bone turnover with centenarian females

As shown in Table 2, height, weight, WC, serum albumin, GNRI, and abdominal obesity were significantly and inversely associated with centenarian females (P < 0.05 for all). Prolactin, progesterone, and estradiol levels were significantly and positively associated with centenarian females (P < 0.05 for all). Osteocalcin, β-crossLaps, and PTH levels were significantly and positively associated with centenarian females (P < 0.05 for all). FSH and 25(OH)D levels were also significantly and inversely associated with centenarian females (P < 0.05 for all).

**Table 2:** Relationships of nutritional status, sex hormones and bone turnover with centenarians in logistic regression

Characteristics	Centenarians		
	Exp(B) (95% CI)	R^2^	P-value
Nutritional status			
Height (cm)	0.959 (0.939–0.980)	0.028	< 0.001
Weight (kg)	0.919 (0.900–0.938)	0.145	< 0.001
Serum albumin (g/dL)	0.059 (0.033–0.104)	0.226	< 0.001
GNRI	0.901 (0.883–0.919)	0.241	< 0.001
Waist circumference (cm)	0.976 (0.960–0.992)	0.017	0.003
Abdominal obesity (%)	0.719 (0.520–0.996)	0.007	0.047
Sex hormones			
LH (mIU/mL)	1.006 (0.995–1.017)	0.002	0.292
FSH (IU/L)	0.994 (0.989–0.999)	0.011	0.015
Testosterone (nmol/L)	0.999 (0.802–1.244)	0.000	0.993
Prolactin (μg/L)	1.073 (1.044–1.103)	0.069	< 0.001
Progesterone (nmol/L)	44.182 (22.036–88.584)	0.348	< 0.001
Estradiol (pmol/L)	1.094 (1.071–1.119)	0.294	< 0.001
Bone turnover			
Osteocalcin (ng/mL)	1.041 (1.028–1.054)	0.090	< 0.001
β-crossLaps (ng/mL)	63.141 (24.482–162.848)	0.181	< 0.001
PTH (pg/mL)	1.022 (1.013–1.031)	0.057	< 0.001
25(OH)D (ng/mL)	0.969 (0.950–0.988)	0.019	0.002

*OR* odds ratio, *CI* confidential interval, *GNRI* geriatric nutritional risk index, *LH* luteinizing hormone, *FSH* follicle-stimulating hormone, *PTH* parathyroid hormone, *25(OH)D* 25-hydroxyvitamin-D3

### Relationships of sex hormones and bone turnover with nutritional status and abdominal obesity

As shown in Table 3, the GNRI was significantly and inversely associated with luteinizing hormone (LH), FSH, progesterone, estradiol, osteocalcin, β-crossLaps, and PTH (P < 0.05 for all). Abdominal obesity was significantly and inversely associated with LH, FSH, osteocalcin, β-crossLaps, and 25(OH)D (P 0.05 for all). Different characteristics between oldest females with and without abdominal obesity are shown in Fig. 1C.

**Table 3:** Relationships of sex hormones and bone turnover with GNRI and abdominal obesity in logistic or linear regression

Characteristics	GNRI			Abdominal obesity		
	β coefficient (95% CI)	R^2^	P-value	Exp(B) (95% CI)	R^2^	P-value
Sex hormones						
LH (mIU/mL)	− 0.001 (− 0.002 to 0.001)	0.199	< 0.001	0.985 (0.974–0.996)	0.024	0.008
FSH (IU/L)	0.000 (− 0.001 to 0.000)	0.186	0.004	0.990 (0.985–0.996)	0.034	0.001
Testosterone (nmol/L)	− 0.005 (− 0.016 to 0.005)	0.178	0.338	1.095 (0.879–1.364)	0.012	0.418
Prolactin (μg/L)	0.000 (− 0.001 to 0.000)	0.177	0.792	1.008 (0.997–1.018)	0.014	0.158
Progesterone (nmol/L)	− 0.016 (− 0.029 to 0.002)	0.182	0.022	1.082 (0.819–1.430)	0.011	0.580
Estradiol (pmol/L)	− 0.001 (− 0.001 to 0.000)	0.205	< 0.001	1.000 (0.994–1.006)	0.010	0.951
Bone turnover						
Osteocalcin (ng/mL)	− 0.001 (− 0.001 to 0.000)	0.187	0.003	0.987 (0.977–0.997)	0.023	0.014
β-crossLaps (ng/mL)	− 0.100 (− 0.130 to 0.071)	0.223	< 0.001	0.338 (0.166–0.689)	0.028	0.003
PTH (pg/mL)	0.000 (− 0.001 to 0.000)	0.188	0.001	0.999 (0.992–1.005)	0.011	0.723
25(OH)D (ng/mL)	0.000 (− 0.001 to 0.001)	0.177	0.411	0.979 (0.960–0.999)	0.018	0.044

*GNRI* geriatric nutritional risk index, *OR* odds ratio, *CI* confidential interval, *LH* luteinizing hormone, *FSH* follicle-stimulating hormone, *PTH* parathyroid hormone, *25(OH)D* 25-hydroxyvitamin-D3

### Relationships between sex hormones and bone turnover

As shown in Table 4, LH levels were significantly and positively associated with osteocalcin, β-crossLaps and PTH and significantly and inversely associated with 25(OH)D (P < 0.05 for all). FSH levels were significantly and positively associated with osteocalcin, β-crossLaps, and PTH (P < 0.05 for all). Progesterone levels were significantly and positively associated with osteocalcin and β-crossLaps (P < 0.05 for all). Estradiol levels were significantly and positively associated with β-crossLaps and significantly and inversely associated with 25(OH)D (P < 0.05 for all).

**Table 4:** Relationships between sex hormones and bone turnover in linear regression

Bone turnover	Osteocalcin			β-crossLaps			PTH			25(OH)D		
Sex hormones	β coefficient (95% CI)	R^2^	P-value	β coefficient (95% CI)	R^2^	P-value	β coefficient (95% CI)	R^2^	P-value	β coefficient (95% CI)	R^2^	P-value
LH (mIU/mL)	0.006 (0.004–0.009)	0.103	< 0.001	0.008 (0.004–0.011)	0.167	< 0.001	0.003 (0.001–0.006)	0.059	0.012	− 0.003 (− 0.004 to 0.001)	0.027	0.008
FSH (IU/L)	0.002 (0.001–0.003)	0.079	0.002	0.003 (0.002–0.005)	0.163	< 0.001	0.002 (0.001–0.003)	0.065	0.001	− 0.001 (− 0.002 to 0.000)	0.021	0.172
Testosterone (nmol/L)	0.031 (− 0.017 to 0.079)	0.068	0.202	0.000 (− 0.066 to 0.065)	0.141	0.991	0.026 (− 0.025 to 0.077)	0.052	0.313	0.022 (− 0.017 to 0.061)	0.020	0.268
Prolactin (μg/L)	− 0.001 (− 0.003 to 0.001)	0.067	0.373	0.002 (− 0.001 to 0.005)	0.143	0.181	0.000 (− 0.002 to 0.003)	0.051	0.902	− 0.001 (− 0.003 to 0.001)	0.020	0.192
Progesterone (nmol/L)	0.124 (0.062–0.185)	0.086	< 0.001	0.095 (0.011–0.179)	0.147	0.027	0.014 (− 0.052 to 0.080)	0.051	0.670	− 0.044 (− 0.094 to 0.006)	0.022	0.083
Estradiol (pmol/L)	0.001 (− 0.001 to 0.002)	0.067	0.405	0.003 (0.001–0.005)	0.155	0.001	0.001 (0.000–0.002)	0.053	0.178	− 0.002 (− 0.003 to 0.001)	0.043	< 0.001

*PTH* parathyroid hormone, *25(OH)D* 25-hydroxyvitamin-D3, *CI* confidential interval, *LH* luteinizing hormone, *FSH* follicle-stimulating hormone

## Discussion

The current study using the CHCCS data demonstrated that centenarian longevity had inverse relationships with nutritional status and abdominal obesity and positive relationships with sex hormones and bone turnover in the oldest females. The current study also confirmed that nutritional status and abdominal obesity had inverse relationships with sex hormones and bone turnover. Moreover, the current study also suggested that sex hormones had positive relationships with bone turnover.

Aging is characterized by a progressive decline in sex function and bone turnover and a continuous increase in nutritional accumulation and body fat [7]. Older females tend to have increased body weights and decreased bone turnover as they proceed beyond menopause [8]. However, compared to older males, older females have less abdominal obesity and are more successful in aging [9]. Some studies regarding age-related changes have evaluated ages up to 90 years but did not analyze the changes correlated with centenarian longevity (≥ 100 years) to obtain information on the factors influencing human longevity [10, 17, 18]. Age-related changes have been reported among older people in Western countries but are not mentioned among the oldest females, including centenarians [10]. The current study provided valuable data on successful age-related changes in the oldest females, including centenarians. The current study found that compared with other oldest centenarians, female centenarians had increased sex hormones and bone turnover and reduced nutritional accumulation and abdominal obesity. Centenarian longevity was inversely related to nutritional status and abdominal obesity and positively related to sex hormones and bone turnover. Excessive nutritional accumulation and obvious abdominal obesity may be detrimental factors of centenarian longevity, while increased sex hormones and enhanced bone turnover may be beneficial factors of centenarian longevity.

Storage and processing methods, especially heat treatments, drive different biochemical reactions and transform the original content of food [19]. This contributes not only to the loss of healthy nutrients but also to the formation of toxic substances [20–22]. Meanwhile, excessive nutritional intake and accumulation obviously accelerate the development of abdominal obesity related to age [23]. Moreover, nutritional requirements change as individuals age; therefore, nutritional intake that has previously been suitable may be superabundant as one ages and could potentially cause abdominal obesity [24]. Abdominal obesity is generally believed to be one of the most severe risk factors of cardiovascular and cerebrovascular diseases, which are primarily responsible for global mortality and are the biggest obstacle to centenarian longevity. Appropriate lifestyle behaviors, such as optimizing nutritional status and avoiding abdominal obesity throughout life, may be the first step in promoting centenarian longevity and successful aging.

In older adults, reduced sex hormone levels compared with those found in young adults have been discussed as an important factor of their frailty [25]. A placebo-controlled study has also suggested beneficial effects of sex hormone replacement therapy on the general well-being of older adults, suggesting that it was an effective and tolerated treatment in postmenopausal women [26]. Although man-made sex hormone supplements may bring about adverse effects, natural sex hormones are beneficial for the well-being of older females. The current study illustrated that female centenarians had higher levels of sex hormones than did those aged between 80 and 99 years; thus, sex hormones may be beneficial factors of centenarian longevity and promote general well-being. Mechanistically, sex hormones have been presumed to play significant roles in improving and maintaining the general well-being of humans, including, but not limited to, nutritional and metabolic regulation, endothelial and immune improvement, and neurotrophic and neuronal remodeling [27, 28]. However, sex hormones are very interesting and complex, especially in the post-menopausal women and oldest females. Further experimental and clinical studies are needed to explain the change in sex hormones in these individuals.

Older people have weaker bone strength and more frequent falls and fractures. Bone loss increases among females around the age of menopause [12]. There is a growing awareness that aging itself accelerates bone loss via mechanisms such as cellular senescence and oxidative stress, which have significant effects on the oldest individuals [29, 30]. However, an increase in bone loss cannot be simply explained by the age-related decline of bone mass [31]. Age-related bone loss and subsequent adverse events are mainly caused by an imbalance in bone formation and absorption [32]. Bone remodeling is characterized by the continuous, orchestrated and cyclic formation and resorption of organic bone matrix [33]. Fundamental changes with aging contribute to an imbalance between bone resorption and formation and to age-related bone loss in adults [34, 35]. The current study documented that female centenarians had higher levels of both bone formation and absorption than those aged 80–99 years. Relatively active and balanced bone formation and absorption may show the vigorous vitality of female centenarians and be significantly beneficial for successful aging. Moreover, centenarian longevity may have significant characteristics of bone turnover with simultaneous increases in bone formation and absorption.

There have been conflicting results from previous studies with respect to the changes in sex hormone levels correlated with nutritional status and abdominal obesity [36]. Some studies have suggested that androgen is actively converted to estrogen in body fat, and therefore, body weight may have a positive correlation with estrogen [37]. However, other studies have reported inverse correlations between sex hormone levels and body weight [38]. Further, studies have indicated positive correlations between sex hormone levels and muscle mass and strength [39]. Sex hormones may reduce the shift from muscle to fat and prevent nutritional accumulation and abdominal obesity [40]. The current study proposed that sex hormones had inverse relationships with nutritional status and abdominal obesity in female centenarians. Increased sex hormone levels in female centenarians may reduce nutritional accumulation and abdominal obesity and promote centenarian longevity and successful aging.

Previous studies have described that bone turnover was significantly correlated with body weight in adults [41]. Increased body weight at the end of adolescence and at the beginning of adulthood is well correlated with increased bone mass [33]. Load mechanisms are imposed by body weight, particularly by lean body mass, rather than by fat mass [42]. Meanwhile, much attention has been paid to a potential biochemical cross-talk between bone metabolism and fat tissue, termed “bone-fat axis.”. Several molecules, such as osteonectin and osteopontin, have been highlighted to have potential role as communication signals between bone and fat tissue. They may be overlooked biochemical players linking bone and body mass in humans [43]. Moreover, fat tissue is a potential source of hematopoietic stem/progenitor cells, and hormones/adipokines may be involved into bone metabolism regulation and bone marrow-derived hematopoietic stem/progenitor cells mobilization in humans [44]. Subcutaneous and visceral concentrations of osteocalcin have been identified to be associated with body mass or abdominal obesity [45]. However, little is known about the relationships between bone turnover and abdominal obesity in the oldest individuals [46]. Meanwhile, although substantial relationships between bone turnover and nutritional status have been recognized in postmenopausal women, there has been a scarcity of data regarding nutrition-associated changes in bone turnover in the oldest population. The current study indicated that bone turnover had inverse relationships with nutritional status and abdominal obesity. In the oldest adults, excessive nutritional intake results in the accumulation of fat mass in the abdomen but not an increase in muscle mass around the skeleton. Fat mass and abdominal obesity may have detrimental impacts on bone turnover, whereas muscle mass and strength may be beneficial for bone turnover.

Sex hormones have been considered to be essential for bone health and have a certain ability to maintain bone balance [47–49]. A recent Mendelian randomization study identified the beneficial effect of estrogen on bone health in adults [50]. However, few studies have been designed to investigate whether sex hormone levels have significant relationships with bone turnover in the oldest population [30]. Moreover, these studies have evaluated only a few sex hormones rather than the entire spectrum. The current study provided evidence that sex hormone levels had positive relationships with bone turnover. The effects of estrogen on bone turnover are mediated at the cellular level through the activation of estrogen receptor ɑ (ERɑ) [51]. ERα is expressed in osteoblasts, chondrocytes, and osteoclasts [52]. Estrogen acting via ERɑ affects bone health both during pubertal growth and in older people [12]. Rodent studies have also shown that classical nuclear ERɑ signaling and nongenomic actions at the cytoplasmic membrane are required for estrogen effects on bone turnover [53]. Previous randomized trials in adults or older people have also shown that androgen alone seems to have no impact on bone turnover [54], which was confirmed in this study. Meanwhile, there remains a lack of evidence regarding the relationships of sex hormone levels with 25(OH)D and PTH in the oldest females [55]. The current study confirmed that estradiol and LH had significant relationships with 25(OH)D, whereas FSH had a significant relationship with PTH.

In conclusion, the current study using the CHCCS data demonstrated the following relationships in the oldest females: (1) centenarian longevity had inverse relationships with nutritional status and abdominal obesity and positive relationships with sex hormones and bone turnover; (2) nutritional status and abdominal obesity had inverse relationships with sex hormones and bone turnover; and (3) sex hormones had positive relationships with bone turnover. Increased sex hormones and bone turnover may be representative of centenarian longevity. Optimizing nutritional status and avoiding abdominal obesity may increase sex hormones and bone turnover and promote centenarian longevity and successful aging.

## Data Availability

In attempt to preserve the privacy of individuals, clinical data will not be shared; the data can be available from authors upon request.
